# The Influence of Low Doses of Zearalenone on Distribution of Selected Active Substances in Nerve Fibers Within the Circular Muscle Layer of Porcine Ileum

**DOI:** 10.1007/s12031-015-0537-2

**Published:** 2015-03-15

**Authors:** Sławomir Gonkowski, Kazimierz Obremski, Jaroslaw Calka

**Affiliations:** 1 Department of Clinical Physiology, Faculty of Veterinary Medicine, University of Warmia and Mazury, Oczapowski Str. 13, Olsztyn, 10-718 Poland; 2 Department of Veterinary Prevention and Feed Hygiene, Faculty of Veterinary Medicine, University of Warmia and Mazury in Olsztyn, Oczapowski Str. 13, 10-718 Olsztyn, Poland

**Keywords:** Zearalenone, Mycotoxins, Nerve fibers, Gastrointestinal tract, Immunohistochemistry, Pig

## Abstract

The aim of this study was to investigate, whether low doses (25 % of no observable adverse effect levels values) of zearalenone (ZEN) can affect the expression of active substances in nerve fibers in the muscular layer of porcine ileum. The study was performed on ten immature pigs divided into two groups: experimental group (*n* = 5), where zearalenone (10 μg/kg body weight) was given for 42 days, and control animals (*n* = 5), where placebo was administered. Fragments of ileum of all animals were processed for single-labelling immunofluorescence technique using the antibodies against vasoactive intestinal peptide, neuronal form of nitric oxide synthase, cocaine and amphetamine regulatory peptide, galanin, pituitary adenylate cyclase-activating peptide-27 and substance P. The number of nerve fibers immunoreactive to particular substances was evaluated by the counting of nerves per observation field (0.1 mm^2^). Low doses of zearalenone caused the clear changes in the expression of substances studied. The number of nerve fibers immunoreactive to the majority of substances increased in experimental animals. The exception was only galanin, the expression of which was less after administration of zearalenone. The obtained results for the first time show that even low doses of zearalenone can affect the nerve fibers in the digestive tract.

## Introduction

Mycotoxins are a large and varied group of naturally occurring fungal toxins, which are found in agricultural food products and harmful to humans and animals. One of them is zearalenone (ZEN), also known as RAL or F-2 mycotoxin, produced by some *Fusarium* species, whose presence has been observed in a wide variety of food products, such as maize, barley, oat, wheat, rice, peas, bananas, and bread (Briones-Reyes et al. 2007; Hussein and Brasel 2001).

Zearalenone mainly binds to estrogen receptors, as well as interacts with steroidogenic enzymes and, hence, has been classified as an endocrine disrupter. This substance causes the functional and morphological changes in the reproductive system in both animals and humans that interfere with reproduction (Obremski et al. 2003; Tiemann and Danicke 2007; Minervini and Dell’aquila 2008). The poisoning of ZEN is accompanied by typical signs of hyperestrogenism (Diekman and Green 1992) and/or hepatoxicity (Pazaiti et al. 2011) and precocious puberty in girls (Massart et al. 2008). Zearalenone can affect not only organisms, in which this toxin is given with the food, but also their litter by development impairing and reduction of size (Young et al. 1990; Schoevers et al. 2012). Moreover, ZEN can play an important role in the promotion of hormone-dependent tumors (Dees et al. 1997; Tomaszewski et al. 1998) and has an immunosuppressive effect on mononuclear cells in human peripheral blood (Berek et al. 2001). Furthermore, this substance shows genotoxic effects (Pfohl-Leszkowicz et al. 1995) and causes dysfunctions of kidneys and blood coagulation and alterations in hematological parameters (Maaroufi et al. 1996). In addition, ZEN results in adverse effects on gut immunity and mucosal cell proliferation, as well as evokes inflammation of the mucous membrane of the digestive tract (Obremski et al. 2005, 2008; Girish et al. 2010).

The knowledge about effects of ZEN on neurons is extremely scanty and limited to the central nervous system. It is known that estrogen receptors are present in the brain, and phytoestrogens cross the blood–brain barrier (Lephart et al. 2000). Previous studies show that ZEN can exhibit the neurotoxicity in the neurons by the decrease of brain calcium-binding protein level (Lephart et al. 2000) and involvement in oxidative stress mechanisms (Venkataramana et al. 2014). Moreover, it is known that other toxins, which (like ZEN) binds to estrogen receptors, may have deleterious effects on brain cells, such as induction of neuron apoptosis (Doi and Uetsuka 2011) or changes in gene expression and influence on the level of brain-derived neurotropic factor (BDNF)—one of the key regulators of brain function (Pan et al. 1999). The influence of ZEN on nervous structures supplying the intestine has not been studied.

**Fig. 1:**
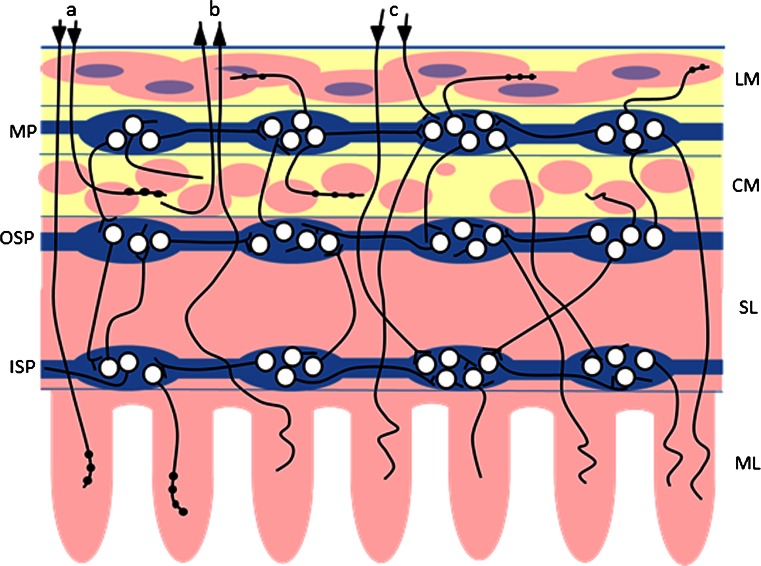
Innervation of the porcine ileum. Enteric nervous system: *MP* myenteric plexus, *OSP* outer submucous plexus, *ISP* inner submucous plexus; extrinsic intestinal innervation: *a* sympathetic postganglionic fibers, *b* sensory fibers, *c* parasympathetic fibers; elements of intestinal wall: *LM* longitudinal muscle layer, *CM* circular muscle layer, *SL* submucosal layer, *ML* mucosal layer.  ascending nerve terminals,  descending nerve terminals

The gastrointestinal (GI) tract is innervated by both the enteric nervous system (ENS) located within intestinal wall and sympathetic, parasympathetic, and sensory extrinsic innervation (Fig. 1) (Gonkowski et al. 2003, 2010; Skobowiat et al. 2010; Ohmori et al. 2012; Wojtkiewicz et al. 2013). It is well known that these neuronal structures, together with intestinal immunological system, are the first line of defense against all damaging factors in the food. These structures are able to undergo changes under pathological stimuli, such as nerve injury, toxic substances, and/or intestinal and extra-intestinal diseases (Vasina et al. 2006; Gonkowski et al. 2003, 2010). Further, changes in expression neuronal factors may be the first subclinical symptoms of pathological processes. However, until now, the influences of ZEN on intestinal neuronal structures have not been studied, although the strong links between estrogen receptors and enteric nervous system (Mathias and Clench 1998; Campbell-Thompson et al. 2001; Bassotti et al. 2012), as well as effects of other mycotoxins on intestinal nervous system (Sousa et al. 2014), suggest that also zearalenone can have considerable influence on nerve structures within the gastrointestinal tract.

Therefore, the aim of the present investigation was the description of ZEN-induced changes in the expression of selected neuronal factors, such as vasoactive intestinal peptide (VIP), neuronal form of nitric oxide synthase (nNOS, that is the marker of nitrergic neurons), cocaine and amphetamine regulatory peptide (CART), galanin (GAL), pituitary adenylate cyclase-activating peptide 27 (PACAP-27), and substance P (SP), within the nerve fibers in circular muscle layer of the porcine ileum, which can come from various parts of nervous system (Fig. 1). The above mentioned substances are known as factors playing important functions within the gastrointestinal tract both in physiological conditions and during various pathological processes, as well as changes in their expression under different stimuli are well documented (Gonkowski et al. 2003, 2010; Vasina et al. 2006; Kaleczyc et al. 2007; Gonkowski and Calka 2012).

Moreover, previous investigations concerning the negative influences of ZEN on living organisms have been mainly performed on high doses of this substance (Liu et al. 2014). In contrast, during the present study, low doses (50 % of no observable adverse effect levels values (NOAEL), i.e., levels at which no clinical symptoms of poisoning are observed) of ZEN has been used and such dosages of zearalenone are most similar to the levels of this toxin to which humans and animals can be exposed in non-laboratory conditions.

It also should be pointed out that the selection of domestic pig to the present investigations is not accidental. This species seems to be an optimal experimental animal for investigations on the gastrointestinal tract because of physiological and pathological similarities between human and porcine enteric nervous system (Brown and Timmermans 2004). Moreover, some authors view the pig as an excellent animal model of various pathological mechanisms in human, such as cardiovascular and gastrointestinal diseases, obesity, diabetes as well as injury and repair tissues (Litten-Brown et al. 2010; Verma et al. 2011).

## Materials and Methods

The present investigation was performed on ten immature female pigs of the Large White Polish breed (approximately 8 weeks old) that were kept under standard laboratory conditions. All treatment on animals were achieved in compliance with the instructions of the Local Ethical Committee in Olsztyn (Poland) (decision number 73/2012/DTN), with special attention paid to minimizing any stress reaction.

The animals were divided into two groups: control (C group; *n* = 5) and experimental (ZEA group; *n* = 5), where zearalenone (Z-0167, Sigma Chemical Co., Steinheim, Germany) with a dose of 10 μg/kg body weight (25 % of NOAEL) (EFSA, 2011) was administered per os in gelatin capsules before the morning feeding everyday for 42 days. At the same time, empty capsules were administered to the control pigs. All animals were euthanized by an overdose of sodium thiopental (Thiopental, Sandoz, Kundl-Rakúsko, Austria) at the end of the experiment (a day after administration of the last dose of ZEN).

After euthanasia, the selfsame fragments of the ileum (ca. 2 cm long) were immediately fixed by immersion in a solution of freshly prepared 4 % buffered paraformaldehyde (pH 7.4) for 30 min rinsed for 72 h in phosphate buffer (0.1 M, pH 7.4, at 4 °C) and transferred into 18 % phosphate-buffered sucrose, where they were kept at 4 °C for at least 5 days until sectioning. Finally, ileal fragments were frozen and fixed on glass slides so that the cutting line was perpendicular to the lumen of gut and cut on a cryostat (−22 °C) into 10-μm-thick sections. These sections were subjected to standard single-labelling immunofluorescence method as described previously by Gonkowski et al. (2009b) using primary antibodies directed towards selected neuronal factors and appropriate secondary reagents (Table 1). Briefly, the staining procedure was as follows: after air-drying at room temperature for 45 min, the sections were incubated with solution containing 10 % of normal goat serum, 0.1 % of bovine serum albumin, 0.01 % of NaN_3_, Triton X-100, and thimerozal in PBS for 1 h at room temperature, then incubated with the primary antiserum (overnight; at room temperature), and further incubated with appropriate species-specific secondary antisera conjugated to alexa fluor 594 or alexa fluor 488 (1 h, at room temperature). Each step of immunolabelling was followed by rinsing the sections with PBS (3 × 10 min, pH 7.4). Standard controls, i.e., pre-absorption of the primary antibodies with appropriate antigens and omission and replacement of primary antibody by non-immune serum, were performed to test antibody and specificity of the method. During pre-absorption tests, sections of the ileum were incubated with “working” dilutions of primary antibodies, which previously were pre-absorbed for 18 h at 37 °C with appropriate antigens (Table 1). These tests, as well as omission and replacement, completely eliminated specific stainings.

**Table 1:** List of antibodies and reagents used in immunohistochemical investigations

Antigen	Code	Species	Dilution/concentration	Supplier
Primary antibodies				
1	2	3	4	5
CART	H-003-61	Rabbit	1:20000	Phoenix, Pharmaceuticals INC, Belmont, CA, USA
GAL	RIN7153	Rabbit	1:4000	Peninsula, San Carlos, CA, USA
nNOS	N2280	Mouse	1:1000	Sigma-Aldrich, Saint Luis, MS, USA
PACAP-27	IHC 8922	Rabbit	1:20000	Phoenix Pharmaceuticals, INC, Belmont, CA, USA
SP	8450-0505	Rat	1:500	Biogenesis Ltd., Poole, England
VIP	VA1285	Rabbit	1:6000	Biomol, Hamburg, Germany
Secondary antibodies and complexes of fluorochromes				
1			4	5
Alexa fluor 488 donkey anti-mouse IgG			1:1000	Invitrogen, Carlsbad, CA, USA
Alexa fluor 488 donkey anti-rat IgG			1:1000	Invitrogen
Alexa fluor 546 donkey anti-rabbit IgG			1:1000	Invitrogen
Antigens used in pre-absorption tests				
1		3	4 (μM)	5
CART		C5977	0.1	Sigma, St. Louis, MO, USA
GAL		G0278	0.5	Sigma
NOS		N3033	1.0	Sigma
PACAP-27		052-02	0.3	Phoenix Pharmaceuticals
SP		S6883	0.7	Sigma
VIP		V6130	1.0	Sigma

The labelled sections were studied with Olympus fluorescence microscope equipped with epi-illumination and appropriate filter sets. Evaluation of the density of nerves within the muscular layer was based on counting all the nerve fibres that were immunoreactive to individual neuronal factor studied per observation field (0.1 mm^2^). Nerve profiles were counted in 20 observation fields in each animal (four sections and five fields per section), and to prevent double counting of nerve fibres, the sections were located at least 500 μm apart from each other. Finally, the obtained data were pooled and expressed as mean ± SEM. Statistical analysis was carried out with Student’s *t* test (Graphpad Prism v. 6.0; GraphPad Software Inc., San Diego, CA, USA). The differences were considered statistically significant at *p* ≤ 0.05.

## Results

**Fig. 2:**
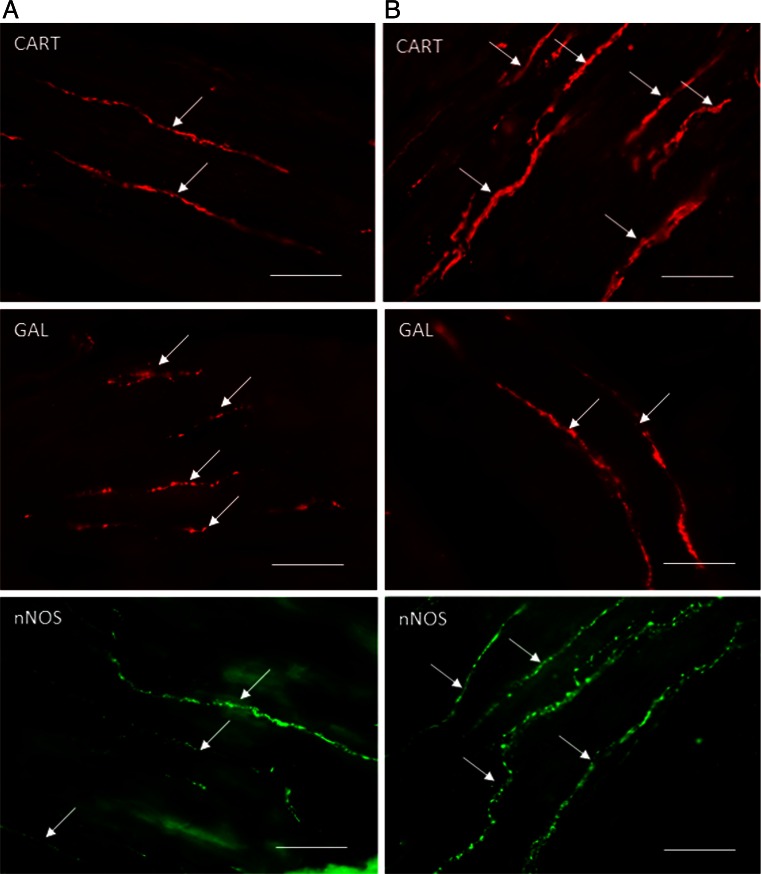
Nerve fibers immunoreactive to cocaine and amphetamine regulatory peptide (*CART*), galanin (*GAL*), and neuronal isoform of nitric oxide synthase (*nNOS*) in the circular muscle layer of the porcine ileum in physiological conditions (**a**) and after administration of zearalenone (**b**). Scale bar 50 μm

**Fig. 3:**
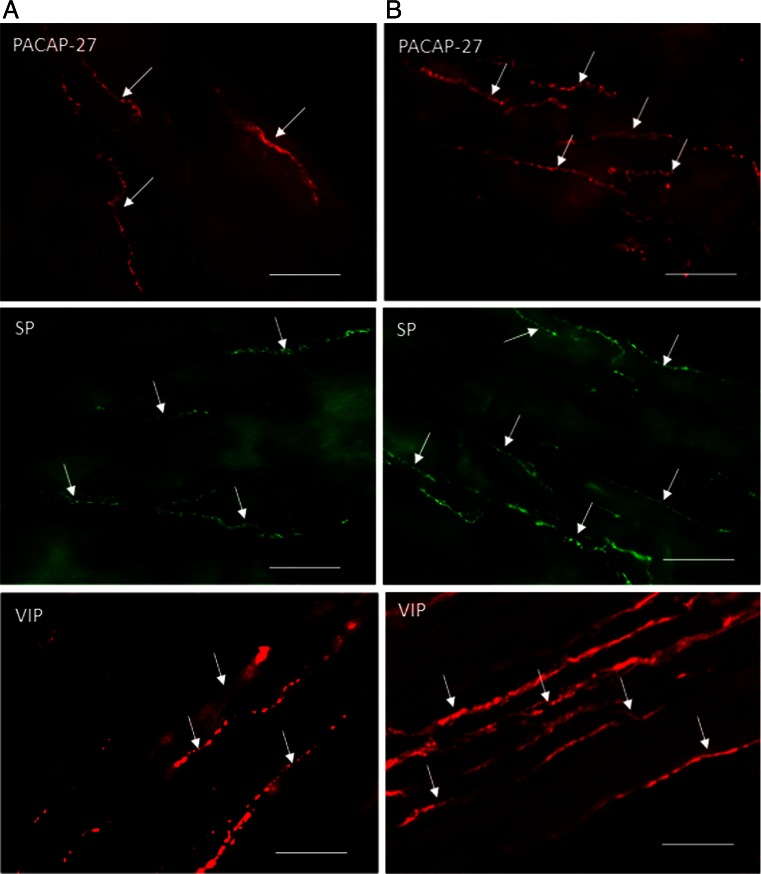
Nerve fibers like immunoreactive to pituitary adenylate cyclase-activating peptide 27 (*PACAP-27*), substance P (*SP*), and vasoactive intestinal peptide (*VIP*) in the circular muscle layer of the porcine ileum in the physiological conditions (**a**) and after administration of zearalenone (**b**). Scale bar 50 μm

During the present investigations, nerve fibers immunoreactive to all active substances studied were observed in the circular muscle layer of the porcine ileum both in physiological conditions and after administration of zearalenone (Table 2 and Figs. 2 and 3).

**Table 2:** Number of nerve profiles immunoreactive to various active substances studied per observation field (0.1 mm^2)^ in the circular muscle layer of porcine ileum in physiological conditions (C group) and after administration of zearalenone (ZEN group) (mean ± SEM)

Substance	C group	ZEA group	Character of changes
CART	14.09 ± 0.20	17.14 ± 0.30	↑
GAL	15.34 ± 0.40	10.84 ± 0.52	↓
nNOS	11.50 ± 0.22	13.60 ± 0.31	↑
PACAP-27	10.37 ± 0.44	14.52 ± 0.49	↑
SP	10.10 ± 0.21	15.48 ± 0.37	↑
VIP	14.33 ± 0.49	19.61 ± 0.54	↑

In control animals, the majority of nerve processes were immunoreactive to CART (Fig. 2), GAL (Fig. 2), and VIP (Fig. 3), and the number of these nerves was rather evenly and fluctuated between about 14 to a little over 15 in one observation field (Table 2). In the event of fibers immunoreactive to other substance studied, a slightly lower density of them was observed during the present investigation (Table 2). Regarding nNOS-immunoreactive nerves (Fig. 2), these values amounted to about 11 and, in the case of SP- (Fig. 3) and PACAP-27-positive nerve processes (Fig. 3), a just over ten nerve fibers per observation field. Moreover, dependences between the appearance of nerve fibers and the type of neuronal factor presented in them were observed. Namely, while nerve processes immunoreactive to CART, GAL, nNOS, PACAP-27, and VIP were thick and built the large, good visible bundles, SP-positive fibers were rather thin and delicate (Figs. 2 and 3).

The administration of low doses of zearalenone caused the statistically significant changes in the number of nerve fibers immunoreactive to all neuronal factors studied, and these changes depended on the type of substance (Table 2). An increase in the number of nerve processes exhibiting the presence of majority of substances studied was observed. The most significant changes are related to VIP- and SP-positive nerves (Fig. 3). A slightly lower increase was observed in the case of processes immunoreactive to PACAP-27 (Fig. 3) and CART (Fig. 2), and the changes in nNOS-positive processes (Fig. 2) were the least clear, but also statistically significant (Table 2).

Contrary to the abovementioned substances, the number of nerve processes immunoreactive to galanin markedly decreased after administration of zearalenone (Fig. 2). The number of GAL-positive fibers, which in control animals was most numerous, in experimental pigs amounted to about ten in observation field and was the least prolific of the processes immunoreactive to all neuronal factor studied (Table 2). In pigs, after administration of zearalenone, VIP- and CART-like immunoreactive nerve fibers were the most numerous (Table 2).

During the present study, visible differences in the appearance of nerve processes immunoreactive to particular neuronal factors between control animals and in pigs after administration of ZEA were not observed (Figs. 2 and 3).

## Discussion

The obtained results exhibiting all neuronal factors studied in nerve fibers within the porcine ileal circular muscle layer confirm the previous studies, where these substances were investigated in the muscular membrane of the digestive tract of human and other mammals (Kaleczyc et al. 2007; Gonkowski et al. 2003; 2009a, b, 2010; Wojtkiewicz et al. 2012).

It is well known that the majority of nerves within muscles of stomach and intestines takes part in the regulation of gastrointestinal motility, and such functions of all studied in the present work substances were previously described. Some of these substances play relatively known functions in the regulation of intestinal motility. One of them is VIP, which is widely distributed in neuronal structures of the GI tract and is known as an inhibitor of smooth muscle contraction (El-Mahmoudy et al. 2006; Kaleczyc et al. 2007; Kasparek et al. 2007). Similar actions are characteristic for PACAP that induces muscle relaxation in the esophagus, stomach, and intestine (Lauffer et al. 2004; Zizzo et al. 2004) and nitric oxide, known as non-adrenergic and non-cholinergic inhibitory factor, which can act on intestinal motility (Grider 1993; Daniel et al. 1994) with diastolic effects within the esophagus, stomach, small and large intestine, as well as in the internal anal sphincter (Murray et al. 1991; Sarna et al. 1993; Schleiffer and Raul 1997).

In contrast to the abovementioned neuronal factors, the influence of GAL and SP on the intestinal motor activity is differential and clearly depends on the part of the GI tract and species studied. Galanin, for example, induces the contraction of the muscles within the ileum of the rat, rabbit, and pig (Botella et al. 1992), while in the same part of canine digestive tract, as well as in the canine pylorus, it evinces diastolic activity (Fox-Threlkeld et al. 1991). Likewise, SP is known as a strong contractive factor within the intestinal muscles of rats and dogs (Lördal et al. 1993; Thor et al. 1982), while in human this function of SP is less important (Lördal et al. 1997).

The most obscure substance of all neuronal factors studied during the present investigation is CART. Numerous intramuscular CART-positive nerve fibers have been describe in the GI tract of various species (Ekblad 2006; Arciszewski et al. 2009; Gonkowski et al. 2009a,b; Wojtkiewicz et al. 2012), but its influence on the gastrointestinal muscles remains unclear. Some studies show that CART is a reducer of intestinal motility via cholinergic pathways (Tebbe et al. 2004), while others suggest systolic activity of this substance by inhibition of nitric oxide-induced muscular relaxation (Ekblad et al. 2003). Nonetheless, previous in vitro investigations on dissected fragments of various parts of the GI tract have not shown any contractile or relaxatory activity of CART (Ekblad et al. 2003).

During the present study, changes in the number of intramuscular nerve fibers immunoreactive to all neuronal factor studied were observed after administration of zearalenone. This fact confirms relatively well-known knowledge that expression of active substances in neuronal structures of the gastrointestinal tract can change under different stimuli, belonging to both physiological and pathological agents (Vasina et al. 2006; Kaleczyc et al. 2007; Gonkowski et al. 2009b, 2010). The character of changes in the expression of neuronal factors depends on their type, part of the GI tract, and, primarily, the kind of stimulus acting. For this reason, the explanation of precise mechanisms of the abovementioned processes is very difficult. Generally, it is accepted that expression of neuronal factors exhibiting the neuroprotective action increases during the majority of pathological stimuli (Arciszewski and Ekblad 2005). Moreover, all neuronal factors studied in the present investigation are known as substances that take part in adaptation of the GI tract during various pathological processes (Vasina et al. 2006).

The present results suggest that also low doses of zearalenone can affect the nervous system within the GI tract. Most likely, it is connected with the action of ZEN on estrogen receptors within the GI tract and, in consequence, on intestinal endocrine system, which closely cooperates with neuronal structures (Mathias and Clench 1998; Campbell-Thompson et al. 2001; Bassotti et al. 2012). Moreover, it is possible that observed changes are also the result of effects of ZEN on gut immune system and its pro-inflammatory properties. Namely, previous studies show that ZEN added to feed influences the cytokine levels produced by Th1 and Th2 subpopulations of lymphocytes in porcine ileal Peyer’s patches. These changes were expressed in significant increase in levels of interleukin-2, interleukin-4, interleukin-10, and interferon-γ and depended on the duration of toxin exposure (Obremski 2014a). Other publication exhibited that the long-term exposure to ZEN at doses below the NOEL threshold (EFSA 2011) can alter the sensitivity of T and B lymphocytes to lipopolysaccharides (LPS) stimulation in vitro, what causes the inhibition of interleukin-2 and interferon-γ production and stimulation of interleukin-4 and interleukin-10 secretion (Obremski 2014b). Moreover, the influences of ZEN on gut immunity and cell proliferation were also described within avian lymphoid organs (Girish et al. 2010).

Nonetheless, the mechanisms of observed changes are unclear. Namely, they can be caused by modifications in the neuronal transport of particular substances, as well as by increase or reduction (in the case of GAL) in expression of them, what, in turn, can be a reflection of changes in the transcriptional, translational, or metabolic level.

To sum up, the obtained results show for the first time that zearalenone can change immunoreactivity of nervous structures in the ileal wall, what can suggest toxic actions of even low doses of this mycotoxin. On the other hand, the mechanism of observed changes remains unknown and requires further investigations.

## References

[CR1] Arciszewski MB, Ekblad E (2005). Effects of vasoactive intestinal peptide and galanin on survival of cultured porcine myenteric neurons. Regul Pept.

[CR2] Arciszewski MB, Barabasz S, Skobowiat C, Maksymowicz W, Majewski M (2009). Immunodetection of cocaine- and amphetamine-regulated transcript in the rumen, reticulum, omasum and abomasum of the sheep. Anat Histol Embryol.

[CR3] Bassotti G, Villanacci V, Bellomi A, Fante R, Cadei M, Vicenzi L, Tonelli F, Nesi G, Asteria CR (2012). An assessment of enteric nervous system and estroprogestinic receptors in obstructed defecation associated with rectal intussusception. Neurogastroenterol Motil.

[CR4] Berek L, Petri IB, Mesterhazy A, Teren J, Molnar J (2001). Effects of mycotoxins on human immune functions in vitro. Toxicol In Vitro.

[CR5] Botella A, Delvaux M, Frexinos J, Bueno L (1992). Comparative effects of galanin on isolated smooth muscle cells from ileum in five mammalian species. Life Sci.

[CR6] Briones-Reyes D, Gómez-Martinez L, Cueva-Rolón R (2007). Zearalenone contamination in corn for human consumption in the state of Tlaxcala,. Mexico Food Chem.

[CR7] Brown DR, Timmermans JP (2004). Lessons from the porcine enteric nervous system. Neurogastroenterol Motil.

[CR8] Campbell-Thompson M, Reyher KK, Wilkinson LB (2001). Immunolocalization of estrogen receptor alpha and beta in gastric epithelium and enteric neurons. J Endocrinol.

[CR9] Daniel EE, Haugh C, Woskowska Z, Cipris S, Jury J, Fox-Threlkeld JET (1994). Role of nitric oxide-related inhibition in intestinal function: relation to vasoactive intestinal polypeptide. Am J Physiol.

[CR10] Dees C, Foster JS, Ahamed S, Wimalasena J (1997). Dietary estrogens stimulate human breast cells to enter the cell cycle. Environ Health Perspect.

[CR11] Diekman MA, Green ML (1992). Mycotoxins and reproduction in domestic livestock. J Anim Sci.

[CR12] Doi K, Uetsuka K (2011). Mechanisms of mycotoxin-induced neurotoxicity through oxidative stress-associated pathways. Int J Mol Sci.

[CR13] EFSA (European Food Safety Authority) (2011). Opinion on the risks for public health related to the presence of zearalenone in food. EFSA J.

[CR14] Ekblad E (2006). CART in the enteric nervous system. Peptides.

[CR15] Ekblad E, Kuhar MJ, Wierup N, Sundler F (2003). Cocaine- and amphetamine-regulated transcript distribution and function in rat gastrointestinal tract. Neurogastroenterol Motil.

[CR16] El-Mahmoudy A, Khalifa M, Draid M, Shiina T, Shimizu Y, El-Sayed M, Takewaki T (2006). NANC inhibitory neuromuscular transmission in the hamster distal colon. Pharmacol Res Dec.

[CR17] Fox-Threlkeld JET, McDonald TJ, Cipris S, Woskowska Z, Daniel EE (1991). Galanin inhibition of vasoactive intestinal polypeptide release and circular muscle motility in the isolated perfused canine ileum. Gastroenterology.

[CR18] Girish CK, Smith TK, Boermans HJ, Anil Kumar P, Girgis GN (2010). Effects of dietary Fusarium mycotoxins on intestinal lymphocyte subset populations, cell proliferation and histological changes in avian lymphoid organs. Food Chem Toxicol.

[CR19] Gonkowski S, Calka J (2012) Changes in pituitary adenylate cyclase-activating Peptide 27-like immunoreactive nervous structures in the porcine descending colon during selected pathological processes. J Mol Neurosci 48:777–787 10.1007/s12031-012-9838-x 22706710

[CR20] Gonkowski S, Kaminska B, Bossowska A, Korzon M, Landowski P, Majewski M (2003). The influence of experimental Bacteroides fragilis infection on substance P and somatostatin-immunoreactive neural elements in the porcine ascending colon—a preliminary report. Folia Morphol.

[CR21] Gonkowski S, Burliński P, Skobowiat C, Majewski M, Arciszewski MB, Radziszewski P, Calka J (2009a) Distribution of cocaine- and amphetamine-regulated transcript-like immunoreactive (CART-LI) nerve structures in the porcine large intestine. Acta Vet Hung 57:509–520 10.1556/AVet.57.2009.4.5 19897455

[CR22] Gonkowski S, Kaminska B, Burlinski P, Kroll A, Calka J (2009). The influence of drug-resistant ulcerative colitis on the number of cocaine- and amphetamine-regulated transcript peptide-like immunoreactive (CART-LI) mucosal nerve fibres of the descending colon in children. Przeglad Gastroenterologiczny.

[CR23] Gonkowski S, Burliński P, Skobowiat C, Majewski M, Calka J (2010). Inflammation- and axotomy-induced changes in galanin-like immunoreactive (GAL-LI) nerve structures in the porcine descending colon. Acta Vet Hung.

[CR24] Grider JR (1993). Interplay of VIP and nitric oxide in regulation of the descending relaxation phase of peristalsis. Am J Physiol.

[CR25] Hussein HS, Brasel JM (2001). Toxicity, metabolism, and impact of mycotoxins on humans and animals. Toxicology.

[CR26] Kaleczyc J, Klimczuk M, Franke-Radowiecka A, Sienkiewicz W, Majewski M, Łakomy M (2007). The distribution and chemical coding of intramural neurons supplying the porcine stomach—the study on normal pigs and on animals suffering from swine dysentery. Anat Histol Embryol.

[CR27] Kasparek MS, Fatima J, Iqbal CW, Duenes JA, Sarr MG (2007). Role of VIP and Substance P in NANC innervation in the longitudinal smooth muscle of the rat jejunum influence of extrinsic denervation. J Surg Res.

[CR28] Lauffer JM, Modlin IM, Tang LH (2004). Biological relevance of pituitary adenylate cyclase activating polypeptide (PACAP) in the gastrointestinal tract. Regul Pept.

[CR29] Lephart ED, Thompson JM, Setchell KDR, Adlercreutz H, Weber KS (2000). Phytoestrogens decrease brain calcium-binding proteins but do not alter hypothalamic androgen metabolizing enzymes in adult male rats. Brain Res.

[CR30] Litten-Brown JC, Corson AM, Clarke L (2010). Porcine models for the metabolic syndrome, digestive and bone disorders: a general overview. Animal.

[CR31] Liu M, Gao R, Meng Q, Zhang Y, Bi C, Shan A (2014). Toxic effects of maternal zearalenone exposure on intestinal oxidative stress, barrier function, immunological and morphological changes in rats. PLoS ONE.

[CR32] Lördal M, Johansson C, Hellström PM (1993). Myoelectric pattern and effects on small bowel transit induced by the tachykinins neurokinin A, neurokinin B, substance P and neuropedtide K in the rat. J Gastrointest Motil.

[CR33] Lördal M, Theodorsson E, Hellström PM (1997). Tachykinins influence interdigestive rhythm and contractile strength of human small intestine. Dig Dis Sci.

[CR34] Maaroufi K, Chekir L, Creppy EE, Ellouz F, Bacha H (1996). Zearalenone induces modifications of haematological and biochemical parameters in rats. Toxicon.

[CR35] Massart F, Meucci V, Saggese G, Soldani G (2008). High growth rate of girls with precocious puberty exposed to estrogenic mycotoxins. J Pediatr.

[CR36] Mathias JR, Clench MH (1998). Relationship of reproductive hormones and neuromuscular disease of the gastrointestinal tract. Dig Dis.

[CR37] Minervini F, Dell’aquila ME (2008) Zearalenone and reproductive function in farm animals. Int J Mol Sci 92570–92584 10.3390/ijms9122570 PMC2635636 19330093

[CR38] Murray J, Du C, Ledlow A, Bates JN, Conkin JL (1991). Nitric oxide: mediator of nonadrenergic noncholinergic responses of opossum esophageal muscle. Am J Physiol.

[CR39] Obremski K (2014). Changes in Th1 and Th2 cytokines concentrations in ileal Peyer’s patches in gilts exposed to zearalenone. Pol J Vet Sci.

[CR40] Obremski K (2014). The effect of in vivo exposure to zearalenone on cytokine secretion by Th1 and Th2 lymphocytes in porcine Peyer’s patches after in vitro stimulation with LPS. Pol J Vet Sci.

[CR41] Obremski K, Gajęcki M, Zwierzchowski W, Zielonka Ł, Otrocka-Domagała I, Rotkiewicz T, Mikołajczyk A, Gajęcka M, Polak M (2003). Influence of zearalenone on reproductive system cell proliferation in gilts. Pol J Vet Sci.

[CR42] Obremski K, Gajęcka M, Zielonka Ł, Jakimiuk E, Gajęcki M (2005). Morphology and ultrastructure of small intestine mucosa in gilts with zearalenone mycotoxicosis. Pol J Vet Sci.

[CR43] Obremski K, Zielonka L, Gajecka M, Jakimiuk E, Bakuła T, Baranowski M, Gajecki M (2008). Histological estimation of the small intestine wall after administration of feed containing deoxynivalenol, T-2 toxin and zearalenone in the pig. Pol J Vet Sci.

[CR44] Ohmori Y, Atoji Y, Saito S, Ueno H, Inoshima Y, Ishiguro N (2012). Differences in extrinsic innervation patterns of the small intestine in the cattle and sheep. Auton Neurosci.

[CR45] Pan Y, Anthony M, Clarkson TB (1999). Evidence for up-regulation of brain-derived neurotrophic factor mRNA by soy phytoestrogens in the frontal cortex of retired breeder female rats. Neurosci Lett.

[CR46] Pazaiti A, Kontos M, Fentiman IS (2011). ZEN and the art of breast health maintenance. Int J Clin Pract.

[CR47] Pfohl-Leszkowicz A, Chekir-Ghedira L, Bacha H (1995). Genotoxicity of zearalenone, an estrogenic mycotoxin: DNA adduct formation in female mouse tissues. Carcinogenesis.

[CR48] Sarna SK, Otterson MF, Ryan RP, Cowles VE (1993). Nitric oxide regulates migrating motor complex cycling and its postprandial disruption. Am J Physiol.

[CR49] Schleiffer R, Raul F (1997). Nitric oxide and the digestive system in mammals and non-mammalian vertebrates. Comp Biochem Physiol.

[CR50] Schoevers EJ, Santos RR, Colenbrandera B, Fink-Gremmelsb J, Roelen BAJ (2012). Transgenerational toxicity of zearalenone in pigs. Reprod Toxicol.

[CR51] Skobowiat C, Gonkowski S, Calka J (2010). Phenotyping of sympathetic chain ganglia (SChG) neurons in porcine colitis. J Vet Med Sci.

[CR52] Sousa FC, Schamber CR, Amorin SS, Natali MR (2014) Effect of fumonisin-containing diet on the myenteric plexus of the jejunum in rats. Auton Neurosci 20. doi:10.1016/j.autneu.2014.08.001 10.1016/j.autneu.2014.08.001 25183308

[CR53] Tebbe JJ, Ortmann E, Schumacher K, Mönnikes H, Kobelt P, Arnold R, Schäffer MKH (2004). Cocaine- and amphetamine-regulated transcript stimulates colonic motility via central CRF receptor activation and peripheral cholinergic pathways in fed conscious rats. Neurogastroenterol Motil.

[CR54] Thor PJ, Sendur R, Konturek SJ (1982). Influence of substance P on myoelectric activity of the small bowel. Am J Physiol.

[CR55] Tiemann U, Danicke S (2007). In vivo and in vitro effects of the mycotoxins zearalenone and deoxynivalenol on different non-reproductive and reproductive organs in female pigs: a review. Food Addit Contam.

[CR56] Tomaszewski J, Miturski R, Semczuk A, Kotarski J, Jakowicki J (1998). Tissue zearalenone concentration in normal, hyperplastic and neoplastic human endometrium. Ginekol Pol.

[CR57] Vasina V, Barbara G, Talamonti L, Stanghellini V, Corinaldesi R, Tonini M, De Ponti F, De Giorgio R (2006). Enteric neuroplasticity evoked by inflammation. Auton Neurosci.

[CR58] Venkataramana M, Chandra Nayaka S, Anand T, Rajesh R, Aiyaz M, Divakara ST, Murali HS, Prakash HS, Lakshmana Rao PV (2014). Zearalenone induced toxicity in SHSY-5Y cells: the role of oxidative stress evidenced by N-acetyl cysteine. Food Chem Toxicol.

[CR59] Verma N, Rettenmeier AW, Schmitz-Spanke S (2011). Recent advances in the use of Sus scrofa (pig) as a model system for proteomic studies. Proteomics.

[CR60] Wojtkiewicz J, Gonkowski S, Bladowski M, Majewski M (2012). Characterization of cocaine- and amohetamine-regulated transcript-like immunoreactive (CART-LI) enteric neurons in the porcine small intestine. Acta Vet Hung.

[CR61] Wojtkiewicz J, Równiak M, Crayton R, Gonkowski S, Robak A, Zalecki M, Majewski M, Klimaschewski L (2013). Axotomy-induced changes in the chemical coding pattern of colon projecting calbindin-positive neurons in the inferior mesenteric ganglia of the pig. J Mol Neurosci.

[CR62] Young LG, Ping H, King GJ (1990). Effects of feeding zearalenone to sows on rebreeding and pregnancy. J Anim Sci.

[CR63] Zizzo MG, Mule F, Serio S (2004). Interplay between PACAP and NO in mouse ileum. Neuropharmacology.

